# Influence of Universal Adhesives With or Without Silane Compared to One‐Step and Two‐Step Silane Agents on Bond Strength to Lithium Disilicate

**DOI:** 10.1111/jerd.70240

**Published:** 2026-07-25

**Authors:** Camilo Pulido, Michel Wendlinger, Gabriel David Cochinski, Camila Farias Monteles, Alessandro Dourado Loguercio

**Affiliations:** ^1^ Department of Oral Health, Facultad de Odontología Universidad Nacional de Colombia Bogotá Colombia; ^2^ Department of Dental System, Facultad de Odontología Pontifícia Universidad Javeriana Bogotá Colombia; ^3^ Department of Restorative Dentistry State University of Ponta Grossa Ponta Grossa Brazil; ^4^ Department of Restorative Dentistry, School of Dentistry Ponta Grossa State University (UEPG) Ponta Grossa Brazil

**Keywords:** adhesives, bond strength, lithium disilicate, silane coupling agents

## Abstract

**Objective:**

To evaluate the microshear bond strength (μSBS) to lithium disilicate achieved with universal adhesives (with and without silane) compared with one‐step and two‐step silanes, immediately (IM) and after thermocycling (TC). Chemical interaction (CI) was also assessed.

**Material and Methods:**

Lithium disilicate blocks were randomly assigned to nine groups (*n* = 10) according to surface treatment (silane‐free [ALB; AllBond Universal] and silane‐containing [AMU; Ambar Universal APS Plus, SBU; Scotchbond Universal Plus] universal adhesives, one‐step [PPR; Porcelain Primer, PRO; Prosil, CPR; Ceramic Primer], or two‐step silanes [BIS; Bis‐Silane, EXP; experimental silane, SIL; Silano Mais]). After hydrofluoric acid etching, treatments were applied and resin cement cylinders bonded. μSBS was measured after 24 h or 10,000 thermocycles. CI was analyzed by micro‐Raman spectroscopy. Data were evaluated using two‐way ANOVA and Bonferroni tests (*α* = 0.05).

**Results:**

Surface treatment, evaluation time, and their interaction significantly influenced μSBS (*p* < 0.001). IM, two‐step silanes, PPR, and AMU showed higher μSBS, whereas the silane‐free universal adhesive demonstrated lower values. After TC, SIL, BIS, PPR, and AMU maintained higher μSBS. Raman analysis demonstrated comparable CI for AMU and SBU compared with one‐ and two‐step silanes.

**Conclusions:**

Bond strength was influenced by pretreatment strategy and aging. Two‐step silanes were more consistent, although selected simplified systems achieved comparable performance.

**Clinical Significance:**

Two‐step silanes remain the most predictable option; however, certain silane‐containing universal adhesives and specific one‐step silanes may offer simplified alternatives when durability limitations are considered.

## 1 Introduction

Glass‐ceramics are widely used in restorative dentistry due to their favorable combination of esthetics, translucency, and mechanical performance [1]. Achieving durable bonding to these substrates is essential for the long‐term success of bonded ceramic restorations [1, 2]. Their silica‐containing microstructure allows chemical interaction (CI) with silane coupling agents following hydrofluoric acid etching, promoting the formation of siloxane bonds between the ceramic surface and resin‐based materials [3, 4]. This combination of micromechanical interlocking and chemical adhesion enhances wettability, interfacial stability, and overall adhesive performance, contributing to improved long‐term performance [5, 6, 7].

Traditional silanization protocols include one‐step or two‐step systems, which differ in their chemical characteristics and clinical handling. Two‐step silanes typically involve a separate hydrolysis or activation step, allowing greater control over silane reactivity, improved hydrolytic stability, and more reliable interaction with glass–ceramic substrates [5]. These systems have consistently demonstrated high bond strength values and improved resistance to hydrothermal degradation [8]. In contrast, one‐step silanes aim to simplify clinical procedures by combining all components into a single solution. However, prolonged contact between silane molecules and solvents, water, and acidic components within these formulations may compromise chemical stability [9]. Such conditions can promote premature hydrolysis or self‐condensation of silane molecules before clinical application, reducing the availability of reactive silanol groups at the ceramic surface [10]. Consequently, siloxane bond formation may become less predictable, potentially affecting the uniformity and long‐term durability of the adhesive interface [9].

Universal adhesives were developed to further reduce clinical complexity by offering a single material capable of bonding to a broad range of substrates, including enamel, dentin, metals, zirconia, and glass–ceramics [11, 12, 13]. Their versatility is largely attributable to functional monomers, such as 10‐methacryloyloxydecyl dihydrogen phosphate (10‐MDP), which enable chemilca interaction with mineralized tissues and oxide‐containing surfaces [14, 15, 16]. When used for bonding glass–ceramics, some universal adhesives incorporate silane directly into their formulation, while others still require a separate silane application step [17, 18, 19, 20].

Silane‐containing universal adhesives aim to streamline the bonding protocol by eliminating the need for an additional silanization step [21, 22, 23]. However, incorporating silane into adhesive formulations containing water, organic solvents, and acidic monomers imposes inherent chemical limitations, as these environments may compromise silane stability and reduce its reactivity at the time of application [9, 22, 24]. Recent evidence suggests that hydrofluoric acid etching followed by a separate silane application provides more predictable and higher bond strengths to glass–ceramics than the use of silane‐containing universal adhesives [21, 25, 26].

Although manufacturers of newer universal adhesives claim improved stabilization within their acidic adhesive formulations [27, 28, 29, 30], the actual effectiveness and long‐term durability of these incorporated silanes remain uncertain and require further investigation. Recent developments in silane‐containing universal adhesives involve more than simply incorporating silane into the adhesive solution [21]. New formulations may include modifications in silane chemistry, stabilizing agents, solvent composition, water content, and filler incorporation, all of which may influence silane availability, interfacial reactivity, adhesive layer stability, and resistance to hydrothermal degradation [25, 26]. Therefore, the bonding performance of these contemporary universal adhesives cannot be predicted solely by the presence of silane or extrapolated from evidence obtained with earlier formulations. These developments underscore the need for studies directly comparing current silanization strategies under standardized experimental conditions to determine whether these formulation advances translate into improved bonding performance and durability.

Thus, this study aimed to evaluate the microshear bond strength (μSBS) of lithium disilicate achieved with universal adhesives with or without silane, compared with different one‐step and two‐step silane agents, both immediately (IM) and after thermocycling (TC). The research hypotheses were that differences in μSBS to lithium disilicate would be observed between silane‐containing and silane‐free universal adhesives and between one‐step and two‐step silane agents, regardless of evaluation time.

## 2 Materials and Methods

### 2.1 Specimen Preparation

Fifty‐two CAD‐CAM blocks (12 × 12 × 6 mm) of lithium disilicate ceramic (IPS e.max CAD, Ivoclar AG, Schaan, Liechtenstein) were used. Each block was sectioned into four rectangular specimens (6 × 6 × 6 mm; *n* = 208) using a low‐speed diamond disc (Isomet, Buehler, Lake Bluff, IL, USA) under continuous water cooling. The specimens were ultrasonically cleaned with distilled water for 15 min and subsequently crystallized in a ceramic furnace (Programat P300, Ivoclar AG, Schaan, Liechtenstein) according to the manufacturer's firing program at 840°C–850°C for 20–31 min.

### 2.2 Experimental Design

One‐hundred eighty lithium disilicate sections were randomly assigned (*n* = 10 per group) into 18 experimental conditions using an online randomization tool (http://www.sealedenvelope.com) prepared by an independent researcher not involved in the experimental procedures. For μSBS evaluation, the specimens were divided according to the following independent variables: (1) based on surface treatment: Universal adhesives (All‐Bond Universal, Bisco, IL, Schaumburg USA, **ALB**, a silane‐free universal adhesive and Ambar Universal APS Plus, FGM Dental Group, Joinville, SC, Brazil, **AMU**; and Scotchbond Universal Plus, Solventum, St. Paul, MN, USA, **SBU**, as silane‐containing universal adhesives); one‐step silanes (Porcelain Primer, Bisco, IL, Schaumburg USA, **PPR**; Prosil, FGM Dental Group, Joinville, SC, Brazil, **PRO**, and Ceramic Primer, Solventum, St. Paul, MN, USA, **CPR**) and two‐step silane (Bis‐Silane, Bisco, IL, Schaumburg USA, **BIS**; Experimental two‐step silane, FGM Dental Group, Joinville, SC, Brazil, **EXP**, and Silano Mais, Dentsply Sirona, Pirassuninga, SP, Brazil, **SIL**) and (2) evaluation time (immediate [IM] or after TC). The composition and application mode are described in Table 1.

**TABLE 1:** Materials used, batch number, composition, pH and application mode.

Product classification	Material [Batch Number]	Composition^a^ [pH]	Application mode
Silane‐Free Universal adhesive	All‐Bond Universal (Bisco, IL, Schaumburg USA) [2000007597]	Bis‐GMA, Ethanol, 10‐ MDP, HEMA [pH = 3.2]	Apply one coat to the surface and air dry to remove excess solvent. Light cure for 10 s.
Silane‐Containing Universal adhesives	Ambar Universal APS Plus (FGM Dental Group, Joinville, SC, Brazil) [140624]	10‐MDP, methacrylic monomers, APS photoinitiator, camphorquinone, silica nanoparticles, ethanol, co‐initiators, stabilizers, silane [pH = 5.1]	Apply two for 20 s (10 s per coat). Apply a gentle air stream for about 10 s until the material stops moving and the solvent is fully evaporated. Light cure for 10 s.
	Scotchbond Universal Plus (Solventum, St. Paul, MN, USA) [9020296]	10‐MDP phosphate monomer, dimethacrylate resins, HEMA, methacrylate‐modified polyalkenoic acid copolymer, fillers, ethanol, water, initiators, silane [pH = 2.7]	Apply two coats for 20 s (10 s per coat). Apply a gentle air stream for about 10 s until the material stops moving and the solvent is fully evaporated. Light cure for 10 s.
One‐step silane	Porcelain Primer (Bisco, IL, Schaumburg USA) [230001686]	Acetone, 3‐(Trimethoxysilyl)propyl‐2‐Methyl‐2‐Propenoic Acid, Acetic Acid [pH = 4–5]	Using a brush, apply one to two thin layers to the surface and wait 30 s. Remove excess with a gentle dry air stream.
	Prosil (FGM Dental Group, Joinville, SC, Brazil) [010620]	3‐methacryoxypropyltrimethoxysilane, ethanol, water. [pH = 5.3]	Apply one coat of silane to surface. Allow to react for 60 s. Remove excess with a gentle dry air stream.
	RelyX Ceramic Primer (Solventum, St. Paul, MN, USA) [NE74058]	3‐methacryloxypropyltrimethoxysilane, ethyl alcohol, water. [pH = 4.6]	Agitate the bottle. Apply one coat of silane to surface. Allow to react for 60 s. Remove excess with a gentle dry air stream.
Two‐step silane	Bis‐Silane (Bisco, IL, Schaumburg USA) [230001214]	Part A: 3‐(Trimethoxysilyl)propyl‐2‐Methyl‐2‐Propenoic Acid Part B: Ethanol, Phosphoric Acid [pH = 4]	Dispense one drop from each of the two bottles (Parts A and B) and mix well and stir. Brush on one to two thin coats to the surface and wait for 30 s. Dry with air syringe.
	Experimental silane^b^ (FGM Dental Group, Joinville, SC, Brazil) [190922]	Part A: methacryoxypropyltrimethoxysilane, Part B: ethanol and water. [pH = 5.1]	Dispense one drop from each of the two bottles (Parts A and B) and mix well and stir. 2. Brush on one to two thin coats to the surface and wait for 30 s. Dry with air syringe.
	Silano Mais (Dentsply Sirona, Pirassuninga, SP, Brazil) [3828000]	Primer: Ethanol and Silane A 174. Activator: Silane, Ethanol and Glacial Acetic Acid. [pH = 4.0]	Place a drop of Silane Primer and Silane Activator inside a clean dappen vessel. Mix the components for 10–15 s. Allow the Primer/Activator mixture to rest for 5 min (do not exceed 10 min). If excessive evaporation of the Primer/Activator mixture occurs, mix new amounts if necessary. Apply a thin layer of Silano Primer/Activator mixture to the surface. Dry carefully in a moisture‐free or accepted air. Apply a second layer of small thickness and air dry.

^a^ Bis‐GMA: bisphenol A‐glycidyl methacrylate; 10‐MDP: methacryloyloxydecyl dihydrogen phosphate; HEMA: 2‐ Hydroxylethyl methacrylate; APS: Advanced Polymerization System (patented by the company).

^b^ The experimental silane was specifically formulated by the manufacturer for research purposes and was not intended for commercial use. The manufacturer had no role in the study design, data collection, data analysis, interpretation of the results, or manuscript preparation. Furthermore, the authors declare that there are no conflicts of interest related to the experimental material.

Sample size calculation was performed using an online calculator (sealedenvelope.com) based on previously reported μSBS values for silane‐treated lithium disilicate after TC (19.7 ± 3.5 MPa) [16]. Considering a significance level of *α* = 0.05, a statistical power of 80%, and a minimum detectable difference of 5 MPa, the calculated minimum sample size was eight ceramic specimens per group. To compensate for potential specimen loss, the sample size was increased by 20%, resulting in ten ceramic specimens per group (*n* = 10).

Additionally, 27 lithium disilicate sections were assigned to 9 experimental conditions (ALB, AMU, SBU, PPR, PRO, CPR, BIS, EXP, and SIL) for chemical interaction analyses only during IM.

### 2.3 Microshear Bond Strength Test (μSBS)

For the μSBS test, lithium disilicate specimens were embedded in polyvinyl chloride (PVC) rings filled with self‐curing acrylic resin (AutoClear, DentBras, Pirassununga, SP, Brazil), leaving the ceramic surface exposed and a standardized height of 3 mm. All ceramic surfaces were polished using #800‐ to #1200‐grit silicon carbide abrasive papers to standardize the substrate surface and were subsequently ultrasonically cleaned. Following this, the exposed surfaces were etched with 10% hydrofluoric acid for 20 s (Condac 10% Porcelain Etch; FGM Dental Group, Joinville, SC, Brazil), rinsed with running water for 30 s, ultrasonically cleaned in distilled water for 180 s, washed with 96% ethanol, and dried with oil‐free air. The nine experimental pretreatments (ALB, AMU, SBU, PPR, PRO, CPR, BIS, EXP, and SIL) were applied strictly according to the respective manufacturer's recommendations. All procedures were performed by a single calibrated and experienced operator.

Eight transparent polyethylene tubes (Tygon Medical Tubing Formulations 54‐HL, Saint‐Gobain Performance Plastics, Akron, OH, USA) with an internal diameter of 0.8 mm and a height of 0.5 mm were positioned on each ceramic surface. Resin cement (Variolink Esthetic LC, Ivoclar AG, Schaan, Liechtenstein) was inserted into the tubes, covered with a Mylar strip, and gently pressed to ensure complete filling. Light curing was performed for 20 s using a light‐emitting diode (LED) polymerization unit calibrated at 1200 mW/cm^2^ (VALO, Ultradent Products, South Jordan, UT, USA), positioned in direct contact with the Mylar strip. Light irradiance was measured at 8‐specimen intervals using a radiometer (Demetron LED Radiometer, Kerr Sybron Dental Specialties; Middleton, WI, USA). All bonding procedures were carried out using 6× magnification loupes.

After bonding, specimens were stored in distilled water at 37°C for 24 h. The Tygon tubes were then carefully removed with a scalpel, exposing the resin cement cylinders. Each bonded area was inspected under a stereomicroscope at 10× magnification, and cylinders presenting bubbles, gaps, or interfacial defects were discarded. Half of the cylinders were tested after 24 h of water storage (immediate), while the remaining half were subjected to 10,000 thermocycles between 5°C and 55°C, with a dwell time of 1 min in each bath [31].

Each PVC ring was fixed in a shear‐testing jig (Odeme Biotechnology, Luzerna, SC, Brazil) and attached to a universal testing machine (IKCL 3‐USB, Kratos, Cotia, SP, Brazil). A 0.2 mm orthodontic wire loop was positioned around the base of each resin cylinder [32], ensuring proper alignment between the bonded interface, wire loop, and load cell, in accordance with established μSBS testing protocols [33]. The test was performed at a crosshead speed of 1 mm/min until failure.

Microshear bond strength values (MPa) were calculated by dividing the maximum load at failure (N) by the bonded area (mm^2^). After testing, all fractured surfaces were examined under an optical microscope (SZH‐131, Olympus, Tokyo, Japan) at 100× magnification and classified as adhesive (failure at the resin‐ceramic interface), mixed (combination of adhesive and cohesive failures), cohesive in resin cement, or cohesive in ceramic. Premature failures were recorded when specimens exhibited porosity or structural defects detectable at 50× magnification, or when spontaneous detachment occurred prior to testing.

### 2.4 Chemical Interaction Analysis for Micro‐Raman Spectroscopy

Three lithium disilicate sections per group were analyzed immediately after surface treatment using a micro‐Raman spectrometer (LabRAM HR Evolution; Horiba Scientific, Tokyo, Japan). The instrumental was calibrated with a silicon standard (520.7 cm^−1^), and spectra were collected with a 20 mW HeNe laser with 632.8 nm wavelength, spatial resolution of ≈3 μm, spectral resolution ≈5 cm^−1^, accumulation time of 30 s, with 2 coadditions, and magnification of 100× (Olympus UK), and a beam diameter of 1 μm in a wavenumber range of 800–4000 cm^−1^ [34, 35].

Baseline spectra were first obtained from untreated lithium disilicate. Each universal adhesive or silane was then applied according to the manufacturer's instructions (Table 1). The only modification for the protocol was that the universal adhesives were not light‐cured after application. Afterward, the specimens were rinsed for 1 min each in distilled water (30 s) and absolute ethanol (30 s) to remove unreacted or physically adsorbed material. Raman spectra were acquired in triplicate from areas randomly selected on each specimen, using a computer‐generated allocation sequence (https://www.sealedenvelope.com/) prepared by an independent researcher not involved in the experimental procedures. The spectra were qualitatively analyzed by considering the chemical shifts and intensity of each peak and then processed for baseline corrections and normalized.

### 2.5 Statistical Analysis

Data distribution was assessed using the Shapiro–Wilk test, and homogeneity of variances was verified with the Levene test. After confirming the assumptions of normality and homoscedasticity, the μSBS values (MPa) were analyzed using parametric statistics. For statistical purposes, the mean μSBS values of all resin‐cement cylinders bonded to the same ceramic specimen were calculated, with the ceramic specimen considered the experimental unit. Thus, a single mean μSBS value per specimen was included in the analysis, avoiding pseudo‐replication. Premature failures were excluded from the primary statistical analysis. A sensitivity analysis including premature failures yielded the same statistical conclusions (data not shown). This approach is supported by previous studies indicating that, when the frequency of premature failures is low, their inclusion or exclusion does not significantly influence the overall bond strength results [36, 37, 38, 39]. A two‐way ANOVA (“substrate treatment” vs. “storage time”) was performed, followed by Bonferroni's post hoc tests, with the significance level set at *α* = 0.05. The CI was only qualitatively evaluated.

## 3 Results

Adhesive failures predominated across all experimental conditions (Table 2). Pretesting failures (PFs) occurred more frequently in BIS, CPR, and SBU after TC; however, these failures accounted for only 3.75% of the specimens. As the percentage of premature failures was low, these specimens were excluded from the statistical analysis.

**TABLE 2:** Number of specimens (%) according to fracture mode for all experimental groups ^a^.

Product classification	Experimental groups	Immediate					After thermocycling				
		A	M	CR	CC	PF	A	M	CR	CC	PF
Universal adhesive without silane	All‐Bond Universal	78 (97.5)	0 (0)	0 (0)	0 (0)	2 (2.5)	77 (100)	0 (0)	0 (0)	0 (0)	3 (3.75)
Universal adhesives containing silane	Ambar Universal APS Plus	80 (100)	0 (0)	0 (0)	0 (0)	0 (0)	79 (98.7)	0 (0)	0 (0)	0 (0)	1 (1.3)
	Scotchbond Universal Plus	79 (98.7)	0 (0)	0 (0)	0 (0)	1 (1.3)	77 (96.2)	0 (0)	0 (0)	0 (0)	3 (3.8)
One‐step silane	Porcelain Primer	80 (100)	0 (0)	0 (0)	0 (0)	0 (0)	79 (98.7)	0 (0)	0 (0)	0 (0)	1 (1.3)
	Prosil	79 (98.7)	0 (0)	0 (0)	0 (0)	1 (1.3)	78 (97.5)	0 (0)	0 (0)	0 (0)	2 (2.5)
	RelyX Ceramic Primer	78 (97.5)	0 (0)	0 (0)	0 (0)	2 (2.5)	77 (96.2)	0 (0)	0 (0)	0 (0)	3 (3.8)
Two‐step silane	Bis‐Silane	80 (100)	0 (0)	0 (0)	0 (0)	0 (0)	78 (97.5)	0 (0)	0 (0)	0 (0)	2 (2.5)
	Experimental silane	80 (100)	0 (0)	0 (0)	0 (0)	0 (0)	79 (98.7)	0 (0)	0 (0)	0 (0)	1 (1.3)
	Silano Mais	80 (100)	0 (0)	0 (0)	0 (0)	0 (0)	78 (97.5)	0 (0)	0 (0)	0 (0)	2 (2.5)

^a^ Fracture modes: A, adhesive; M, mixed; CR, cohesive in resin cement; CC, cohesive in ceramic; PF, premature failure.

The two‐way ANOVA revealed that μSBS was significantly affected by surface treatment (*p* < 0.001) and aging condition (*p* < 0.001), with a significant interaction between these factors (*p* < 0.001). This interaction suggests that the effect of the pretreatment protocol depended on the evaluation time. Mean μSBS values, standard deviations, and statistical groupings are presented in Table 3.

**TABLE 3:** Mean ± standard deviation of microshear bond strengths (MPa) for all experimental groups.

Product classification	Experimental groups	Immediately	After thermocycling
Universal adhesive without silane	All‐Bond Universal	19.7 ± 3.7 B	12.3 ± 3.3 D
Universal adhesives containing silane	Ambar Universal APS Plus	23.6 ± 4.2 A	21.4 ± 3.3 AB
	Scotchbond Universal Plus	21.6 ± 4.3 AB	19.3 ± 3.9 B
One‐step silane	Porcelain Primer	25.3 ± 3.3 A	21.5 ± 3.6 AB
	Prosil	20.1 ± 3.8 B	15.2 ± 3.1 C
	RelyX Ceramic Primer	18.9 ± 3.5 BC	16.1 ± 3.4 BC
Two‐step silane	Bis‐Silane	25.7 ± 5.2 A	22.6 ± 4.5 AB
	Experimental silane	24.3 ± 4.7 A	18.9 ± 3.6 B
	Silano Mais	24.5 ± 4.4 A	23.2 ± 4.7 A

*Note:* Different letters indicate statistically significant differences among groups (Two‐way ANOVA, Bonferroni's test; *p* < 0.05).

In the immediate condition, pretreatment protocols based on dedicated silane application generally yielded higher μSBS values than the silane‐free universal adhesive (*p* < 0.05). Two‐step silane agents showed the highest immediate bond strength values, with BIS (25.7 ± 5.2 MPa), SIL (24.5 ± 4.4 MPa), and EXP (24.3 ± 4.7 MPa) all classified within the top statistical group. Within the one‐step silane category, performance was more heterogeneous. PPR achieved immediate μSBS values comparable to those of the two‐step silane agents (25.3 ± 3.3 MPa; *p* > 0.05), whereas PRO (20.1 ± 3.8 MPa) and CPR (18.9 ± 3.5 MPa) showed lower immediate values (*p* < 0.05).

Among the universal adhesives, the silane‐containing formulation AMU exhibited high immediate μSBS values (23.6 ± 4.2 MPa), which did not differ statistically from the best‐performing silane protocols (*p* > 0.05). SBU showed intermediate immediate μSBS values (21.6 ± 4.3 MPa), while the silane‐free universal adhesive ALB presented significantly lower immediate μSBS values (19.7 ± 3.7 MPa) compared with most silane‐based pretreatments (*p* < 0.05).

After TC, differences among pretreatment protocols became more pronounced. The two‐step silane agent SIL (23.2 ± 4.7 MPa) exhibited the highest μSBS after aging, followed by BIS (22.6 ± 4.5 MPa), PPR (21.5 ± 3.6 MPa), and AMU (21.4 ± 3.3 MPa), all of which were statistically comparable (*p* > 0.05), indicating greater resistance to TC. The EXP and SBU showed intermediate μSBS values after aging, whereas PRO and CPR exhibited further reductions (*p* > 0.05). Consistently, the silane‐free universal adhesive ALB showed the lowest μSBS values after TC (12.3 ± 3.3 MPa), which were significantly lower than those of all other pretreatment protocols (*p* < 0.05). Overall, TC led to a general reduction in μSBS values (*p* < 0.05); however, the magnitude of this reduction was material dependent.

Representative micro‐Raman spectra of lithium disilicate after different surface treatments are shown in Figure 1. Characteristic Si—O—Si and Si—O stretching bands were clearly identified at approximately 450 and 1100 cm^−1^, respectively, confirming the presence of siloxane bonds on the ceramic surface [40]. A reduction in the intensity of these bands was observed after all surface treatments, suggesting chemical interaction with the lithium disilicate substrate.

**FIGURE 1:**
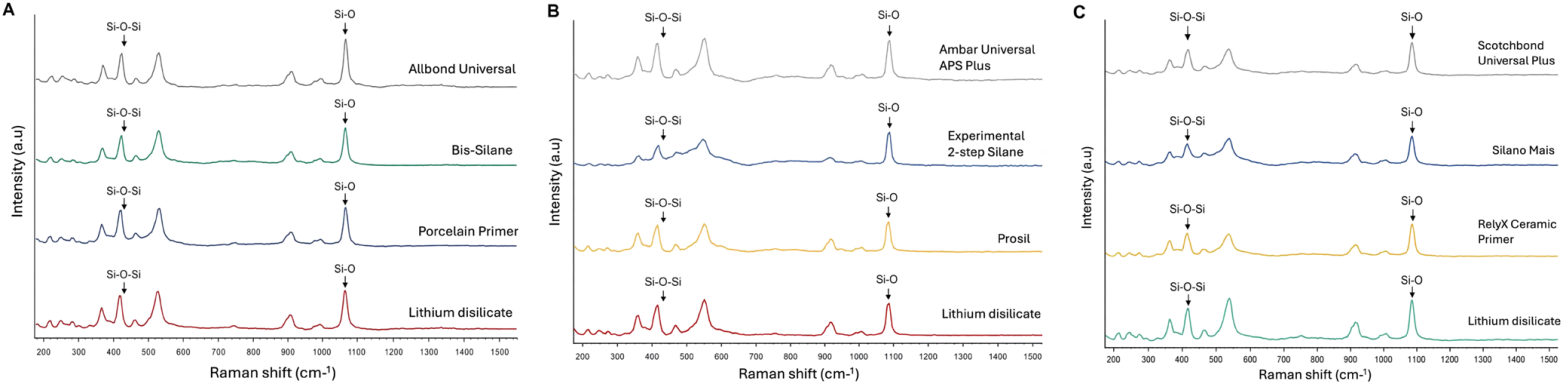
Representative micro‐Raman spectra of lithium disilicate after surface treatments and rinsing illustrating the chemical interaction potential of each treatment. For all spectra (A–C), the first spectrum (from bottom to top) corresponds to the untreated ceramic surface, in which the characteristic Si—O—Si and Si—O stretching bands are clearly identified at approximately 450 and 1100 cm^−1^, respectively. The second, third, and fourth spectra (from bottom to top) represent the ceramic surface after application of the one‐step silane, two‐step silane, and universal adhesive, respectively. Chemical interaction with lithium disilicate is evidenced by the reduction in intensity of the characteristic siloxane‐related peaks (Si—O—Si and Si—O), indicating interfacial bonding with the ceramic substrate. A, includes Bis‐Silane, Porcelain Primer, and All‐Bond Universal; B includes Prosil, Experimental Two‐Step Silane, and Ambar Universal APS Plus; and C includes Silano Mais, RelyX Ceramic Primer, and Scotchbond Universal Plus.

The universal adhesives AMU and SBU exhibited more pronounced spectral changes than ALB, suggesting greater modification of the lithium disilicate surface. Among the silane coupling agents, the two‐step silanes (BIS, EXP, and SIL) exhibited more evident spectral changes on lithium disilicate than the one‐step silanes (PPR, PRO, and CPR). When compared with the silane coupling agents, AMU and SBU exhibited spectral changes of similar magnitude to those observed for both one‐step and two‐step silanes.

## 4 Discussion

The present study investigated the effects of various silane‐based pretreatment strategies and universal adhesives on the μSBS to lithium disilicate under immediate and thermocycled conditions. The results demonstrated that both the pretreatment protocol and aging significantly affected bond strength. Therefore, the research hypotheses were accepted.

The superior performance of two‐step silane agents observed in both evaluation periods aligns with previous evidence reported in the literature [9, 21]. Earlier studies [5, 7] have shown that controlled silane hydrolysis enhances the formation of stable siloxane bonds with silica‐based ceramics, thereby increasing bond strength and resistance to hydrolytic degradation. More recent laboratory investigation has further confirmed that two‐step silane systems tend to offer more reliable and durable adhesion to glass–ceramics than simplified one‐bottle formulations, especially after artificial aging [8]. The findings of the present study support these observations, as the two‐step silane agents consistently exhibited high bond strength values and greater resistance to TC.

Importantly, the micro‐Raman analysis provided complementary evidence supporting these mechanical findings. The characteristic Si–O–Si and Si–O stretching bands of lithium disilicate, identified at approximately 450 and 1100 cm^−1^, respectively, were reduced in intensity after surface treatment. This reduction indicates CI between the applied materials and the ceramic substrate, likely reflecting siloxane bond formation or modification of the silica network. Notably, the spectral pattern observed for the two‐step silanes was more consistent with surface chemical modification of lithium disilicate than that observed for the one‐step silanes. This findings are consistent with the superior bond strength performance observed for the two‐step silane systems and support the concept that optimized silane hydrolysis and condensation contribute to the quality and long‐term stability of the ceramic–resin interface.

In contrast, the performance of the one‐step silane agents evaluated in this study was inconsistent. Although PPR, PRO, and CPR all contain the same functional silane molecule, only PPR achieved bond strength values comparable to those of the two‐step silane systems. These findings suggest that the performance of one‐step silanes depends not only on the silane molecule itself but also on formulation‐related factors, including solvent composition, water content, acidity, and storage stability [9, 30]. These variables may affect the stability of prehydrolyzed silanes and the availability of reactive silanol groups at the time of application [10, 12, 13], thereby influencing the quality of silanization and helping explain the different behavior observed among the one‐step silanes tested. Consequently, formulation‐dependent factors may explain the different bonding performance observed among the one‐step silanes evaluated in the present study.

The positive performance of PPR may be due to its acetone‐based formula combined with mild acidic activation, which is thought to promote quick solvent evaporation and effective silane activation during application. Studies on acetone‐based silane systems have shown improved wetting and more efficient silane deposition on etched ceramic surfaces [29, 30], supporting the findings seen in this study. Conversely, one‐step silanes made mostly with alcohol and water have been linked to reduced stability during storage and higher vulnerability to hydrolytic degradation, which can lead to less consistent bonding results.

Regarding universal adhesives, the current results align with previous studies indicating that adding silane to adhesive formulas can provide sufficient immediate bond strength to glass–ceramic substrates [4, 20, 22, 29]. However, the evidence remains inconclusive about the long‐term effectiveness of silane‐containing universal adhesives. Some authors [25, 27] reported that silane in universal adhesives might be less effective than applying a separate silane treatment, especially after aging. These findings were later supported by a systematic review and meta‐analysis showing that hydrofluoric acid etching followed by a dedicated silane application generally produces stronger bond strength compared to using silane‐containing universal adhesives [21].

The micro‐Raman findings provide additional insight into these results. The universal adhesives AMU and SBU showed spectral changes consistent with surface chemical modification of lithium disilicate, whereas these changes were less evident for the silane‐free adhesive. Moreover, the spectral patterns observed for AMU and SBU were qualitatively similar to those observed for both one‐step and two‐step silanes. These findings suggest that, when appropriately formulated, silane‐containing universal adhesives may induce surface modifications comparable to those produced by dedicated silane coupling agents. However, despite these qualitative spectral similarities, bond durability differed between AMU and SBU after TC, indicating that long‐term bonding performance depends not only on the presence of silane but also on the overall adhesive composition [26, 30].

Differences in adhesive composition may have contributed to the distinct behavior observed between AMU and SBU after TC. Factors such as hydrophilicity, solvent composition, water content, and HEMA concentration have been associated with adhesive permeability, water sorption, and hydrolytic degradation. Therefore, even when similar surface modifications are suggested by micro‐Ramana analysis, long‐term bond durability may still depend on the overall physicochemical characteristics of the adhesive formulation [27].

The lower bond strength values observed for the silane‐free universal adhesive are consistent with previous studies demonstrating that functional monomers, such as 10‐MDP alone, are insufficient to achieve durable adhesion to silica‐based ceramics. Several authors have emphasized that chemical coupling via siloxane bond formation remains essential for achieving stable, long‐lasting bonding to glass–ceramic substrates, regardless of the adhesive strategy employed [3, 12, 16, 23].

Overall, the present findings align with the current body of evidence, indicating that while simplified bonding approaches may provide acceptable immediate performance, long‐term bond durability to glass–ceramics is strongly influenced by silane chemistry and formulation stability. Two‐step silane systems demonstrated greater overall consistency; however, certain one‐step silanes and silane‐containing universal adhesives exhibited bond strength values comparable to those of two‐step silane systems. These findings suggest that bonding performance to glass–ceramics mainly depends on material formulation rather than the number of application steps, and that simplified methods can be effective when properly formulated.

Although the findings of this in vitro study cannot be directly extrapolated to clinical conditions, they reinforce the central role of silanization in achieving and maintaining reliable bond strength to glass‐ceramic substrates over time, highlighting that appropriate silane selection and formulation remain critical even when modern universal adhesives are used. Nevertheless, clinical studies are needed to confirm whether these laboratory observations are maintained under oral environment. In addition, only one lithium disilicate ceramic was evaluated, and differences in ceramic composition, including hybrid or resin‐infiltrated ceramics, may influence the efficacy of silanization and the performance of universal adhesives. Furthermore, the ceramic specimens were polished before hydrofluoric acid etching to standardize the substrate surface, minimizing variability related to milling direction, surface defects, and initial roughness. Although this approach improved experimental standardization, it does not fully reproduce the intaglio surface of CAD/CAM restorations after milling. Therefore, future studies should investigate whether similar results are obtained using clinically milled ceramic surfaces.

## 5 Conclusions

Based on the findings of this in vitro study, the following conclusions were drawn:

1. Both the pretreatment strategy and aging significantly influenced bond strength to lithium disilicate glass‐ceramic substrates.
2. Two‐step silane systems showed greater overall consistency; however, selected one‐step silanes and silane‐containing universal adhesives achieved bond strength and durability comparable to those of conventional systems to lithium disilicate.
3. Bonding performance to lithium disilicate glass‐ceramic appeared to depend primarily on material formulation rather than on the number of application steps alone. Proper silane selection and formulation remain critical for achieving reliable and durable adhesion, even when simplified bonding approaches or modern universal adhesives are used.

## Funding

This work was supported by the National Council for Scientific and Technological Development (CNPq) under grants 304444/2025‐1, as well as the Coordenação de Aperfeiçoamento de Pessoal de Nível Superior, Brazil (CAPES), Finance Code 001.

## Conflicts of Interest

Prof. Alessandro Loguercio is an Associate Editorial of the Journal of Esthetic and Restorative Dentistry. However, he had no involvment on the peer review of this article and had no access to infomation regarding its peer‐review process. The other authors declare no conflicts of interest.

## Data Availability

The data that support the findings of this study are available from the corresponding author upon reasonable request.
